# Incidence of Central Venous Catheter-Related Bloodstream Infections: Evaluation of Bundle Prevention in Two Intensive Care Units in Central Brazil

**DOI:** 10.1155/2019/1025032

**Published:** 2019-10-07

**Authors:** Thais Yoshida, Ana Elisa Bauer de Camargo Silva, Luciana Leite Pineli Simões, Rafael Alves Guimarães

**Affiliations:** ^1^Hospital de Doenças Tropicais Dr. Anuar Auad, Secretaria da Saúde do Estado de Goiás, Goiânia, Goiás, Brazil; ^2^Faculty of Nursing, Federal University of Goiás, Goiânia, Goiás, Brazil; ^3^Institute of Tropical Pathology and Public Health, Federal University of Goiás, Goiânia, Goiás, Brazil

## Abstract

**Background:**

Central venous catheter-associated bloodstream infections (CVC-BSIs) have been associated with increased length of hospital stay, mortality, and healthcare costs, especially in intensive care units (ICUs). The aim of this study was to evaluate the incidence density of CVC-BSIs before and after implementation of the bundle in a hospital of infectious and dermatological diseases in Central Brazil.

**Methods:**

A retrospective cohort study was conducted in two ICUs (adult and pediatric) between 2012 and 2015. Two periods were compared to assess the effect of the intervention in incidence density of CVC-BSIs: before and after intervention, related to the stages before and after the implementation of the bundle, respectively.

**Results:**

No significant reduction was observed in the incidence density of CVC-BSIs in adult ICU (incidence rate ratio [IRR]: 0.754; 95.0% CI: 0.349 to 1.621; p-value = 0.469), despite the high bundle application rate in the postintervention period. Similarly, significant reduction in the incidence density in pediatric ICU has not been verified after implementation of the bundle (IRR: 1.148; 95.0% CI: 0.314 to 4.193; p-value = 0.834).

**Conclusion:**

Not significant reduction in the incidence density of CVC-BSIs was observed after bundle implementation in ICUs, suggesting the need to review the use of process, as well as continuing education for staffs in compliance and correct application of the bundle. Further studies are needed to evaluate the effect of bundle in the reduction of incidence density of CVC-BSIs in Brazil.

## 1. Introduction

Healthcare-associated infections (HAIs) are a serious public health problem and represents significant adverse events in hospitalized patients, especially in intensive care units (ICUs) [1–3]. Central venous catheter-associated bloodstream infections (CVC-BSIs) are among the most serious HAIs and has been associated with increased length of hospital stay, mortality and healthcare costs [4].

It is estimated to occur, per year, in the United States of America (USA), 80.000 cases of CVC-BSIs in ICUs [5]. In addition, in 2013, the National Healthcare Safety Network (NHSN) estimated an incidence of 1.2 BSIs/1,000 CVC-days in medical ICUs in the USA [3]. In European countries, the incidence/1,000 CVC-days range from 1.2 in France to 4.2 in England [2]. In developing countries, especially those in Latin America, the dimension of CVC-BSIs is little known. However, studies conducted in Brazil indicate that the incidence density of CVC-BSIs in ICUs patients has decreased over the years [6]. In 2014, it was recorded an incidence of 5.1 CVC-BSIs/1,000 CVC-days in adult ICUs in Brazil, rate lower than in 2011 (5.9 CVC-BSIs/1,000 CVC-days) [6]. In pediatric ICUs, the incidence density of CVC-BSIs/1,000 CVC-days decreased from 7.3 in 2011 to 5.8 in 2014 [6].

The CVC-BSIs are serious infections and can be prevented through appropriate techniques for insertion and management of CVC [4]. The application of preventive measures in an integrated manner, structured and systemized has shown positive results in reducing these infections and help to increase patient safety in healthcare services [4, 7, 8]. In this context, are bundles of prevention, defined as a set of preventive practices based on evidence that must be performed collectively. The use of these measures allows the evaluation of programs of care and handling of the CVC, to identify potential failures and/or successes that affect the final results. Also enables the calculation of indicators that show the care practice, called process indicators. Care implied in care processes and evaluated through the use of bundles are essential to improving quality and safety in patient care [9, 10].

Several studies have shown decrease in the incidence of CVC-BSIs after bundle implementation [10–15]. A meta-analysis that examined the impact of bundles showed a significant reduction in the median incidence CVC-BSIs after application of these strategies (6.4/1,000 CVC-days versus 2.5/1,000 CVC-days; p-value < 0.001) [10]. The impact of bundles in reducing the incidence of CVC-BSIs depends on multidisciplinary teamwork, effective communication, setting daily goals easily measurable care, continuous professional training, and auditing processes [8]. Thus, despite the positive results in decreasing CVC-BSIs after implementing reported bundles in several investigations, some studies have shown no reduction in CVC-BSIs rates in places like USA, Taiwan, Spain, and Brazil, even after systematic application of these strategies [10].

In Brazil, few studies have investigated the effect of bundle in reducing CVC-BSIs in ICU, and most of these were conducted in the most developed region of the country (Southeast) [13, 14]. Still, studies in pediatric ICU are scarce in Brazil. Thus, this research aimed to evaluate the incidence density of CVC-BSIs before and after implementation of the bundle in a hospital of infectious and dermatological diseases in Central Brazil.

## 2. Materials and Methods

This is a retrospective cohort study that examined the incidence density of CVC-BSIs before and after implementation of bundle of prevention. The research was conducted in adult and pediatric ICUs of a hospital of infectious and dermatological diseases in Central Brazil, from January 2012 to December 2015.

The hospital provides care elective and emergency medium and high complexity exclusively to patients of the Health Unic System (*Sistema Único de Saúde *in Portuguese) in Brazil. This institution has 130 beds distributed in five sectors, two of them in intensive care. The adult ICU has nine beds, four of them for individual isolation of patients, while the pediatric ICU has four hospital beds, two intended for isolation of patients in special care. Overall, they have 100% occupancy rate in all periods. The service profile in both ICUs is for patients with infectious diseases, including Acquired Immune Deficiency Syndrome (AIDS), tuberculosis, meningitis, dengue, among others. Patients are mostly immunosuppressed with use of antimicrobials for community infections, opportunistic or related to health care (HAIs).

The data of this research were obtained by searching the electronic files of Hospital Infection Control Service of the institution, sector responsible for monitoring CVC-BSIs in the ICU. The information files were drawn about the bundle of prevention (components of the package and the total number of applications-days) number of patients-days, number of patients with CVC-days, number of episodes of HAIs, number of cases of CVC-BSIs, number of deaths from CVC-BSIs, and characteristics of patients with CVC-BSIs (age, sex, length of stay, time of use of CVC, diagnosis, and isolated microorganisms).

The study included all cases of CVC-BSIs diagnosed in adult and pediatric ICUs during the analysis period. The case definition was based on criteria established by the National Health Surveillance Agency of Brazil, which are based on the NHSN [16]. The IPCS were defined based on laboratory criteria, that is, diagnosed using blood cultures. Thus, CVC-BSIs were considered if one of the three criteria was met: (i) Criterion 1: patient with one or more positive blood cultures collected preferentially from peripheral blood with the pathogen being not related to infection elsewhere; (ii) Criterion 2: at least one of the following signs or symptoms: fever (> 38°C), tremor, oliguria (urinary volume < 20 ml per hour), and hypotension (systolic pressure ≤ 90 mmHg), these symptoms being unrelated to infection (e.g., diphtheroids,* Bacillus* spp.,* Propionibacterium* spp., staphylococci coagulase negative, and micrococci), or (iii) Criterion 3: for children > 28 days and < 1 year—at least one of the following signs and symptoms: fever (> 38°C), hypothermia (< 36°C), bradycardia or tachycardia (not related to infection elsewhere), and two or more blood cultures (in different punctures with a maximum interval of 48 hours) with common skin contaminants (e.g., diphtheroids,* Bacillus* spp.,* Propionibacterium* spp., staphylococci coagulase negative, and micrococci) [16].

The bundle of prevention of CVC-BSIs was systematically implemented in the institution from September 2014 (adult ICU) to November 2014 (pediatric ICU). It consisted of actions to be performed in all patients using CVC defined from the Institute's recommendations for Health care Improvement (IHI) [17]. This corresponds to an audit tool in the use of the CVC process that consists of four check items in the form of checklists, which are actions to be performed daily in all patients using CVC. The bundle includes the following elements.

- Care in catheter insertion: aseptic technique for catheter insertion (barrier maximum precautions), hand hygiene with chlorhexidine degermante 2%, patient skin antisepsis with degermante 2% chlorhexidine followed by alcoholic 0.5%, and record in the catheter insertion of records with justification statement.
- Care in the administration of drugs: aseptic guns and connections with 70% alcohol before medications and exclusive route for infusion of blood derivatives or parenteral nutrition.
- Care in maintaining the catheter: daily medical records to assess the insertion site; clean, dry dressing and adhered and dated; exchange of catheters inserted in emergency situations and those from other institutions for a maximum of 48 hours; exchange for the infusion system every 96 hours and/or in case of suspicion of pyrogenic shock, and blood visible stuck inside the system; record (date and signature) installation in equipos infusion.
- Daily assessment for early catheter removal: removal of the catheter so that there is no more indication of use, or in the presence of signs and symptoms of catheter-related infection and evidence of medical records of catheter removal with justification statement.

In this study, two periods were compared to assess the effect of the bundle: before intervention (reference period) and after intervention [10]. The preintervention phase encompassed the period from January 2012 to August 2014, in the pediatric ICU, and from January 2012 to October 2014, in the adult ICU, and represented the period before the application of the bundle. Thus, the phase of postintervention contemplated November 2014 to December 2015 in the adult ICU and from September 2014 to December 2015 in the pediatric ICU and reflected the period after implementation of the bundle. The primary outcome was the incidence density of CVC-BSIs preintervention phase compared with the phase of intervention.

Data analysis of adult and pediatric ICU was performed separately. Initially the calculations of process indicators and their respective confidence intervals of 95% (95.0% CI) for each study period were performed. For analysis of the indicator for the bundle, the bundle was calculated application rate, the following formula:

- total number of applications-days bundle in the period by the total number of patients with CVC-days.

For analysis of outcome indicators the following formulas were used:

- Incidence density of HAIs: number of episodes of HAIs in the period by the number of patients-days x 1,000.
- CVC utilization (%): number of patients with CVC-days by the number of patients-days.
- Incidence density of CVC-BSIs number of new cases of CVC-BSIs in the period by the number of patients with CVC-days x 1.000.
- Lethality with CVC-BSIs: number of deaths from CVC-BSIs in the period by the number of patients who developed CVC-BSIs.

Analyses were performed using the Stata software, version 14.0 [18]. The indicators found in before and after intervention were compared using the Wald statistic. For the primary outcome (Incidence density of CVC-BSIs) the bundle effect was analysed using Poisson regression models with robust variance [19]. The models were adjustment by baseline severity. In addition, we included a dummy variable in the model representing intervention “0” in the preintervention period and “1” in the postintervention period [20]. The following assumptions of the Poisson regression were met for model validation: (ii) Independence of observations: it was verified by comparing errors based on standard models with robust errors to determine large differences—for adult ICU the difference of errors between the standard and robust models for the two models was 4.5% and for the pediatric ICU it was 3.4%, suggesting independence; (ii) distribution following a classical Poisson distribution, verifying that the observed and expected data were similar—this assumption was verified by predicting the mean values of the dependent variable and compared by the t test with the observed values [21]—for the adult ICU the observed and expected values were similar (t = 0.000; df: 47; p value = 1.000), as observed in the pediatric ICU model (t = 0.000; df: 47; p value = 1.000) and (iii) the mean and variance of the model are the same or similar, as assessed by Pearson's chi-square of Pearson's chi-square for the pediatric ICU model with a value of 1.067 and for the pediatric ICU model a chi-Pearson square with a value of 1.078, indicating small overdispersion of the data (values less than 1 indicate subdispersion, equal to 1 equidispersion and greater than 1 overdispersion), therefore not causing serious problems in the model. In addition, to reinforce data suitability to Poisson models, the goodness-of-fit Deviance was performed - the result for the adult ICU was a chi-square of 53.70 (df: 45; p value = 0.175) and for pediatric ICU a chi-square of 28,621 (df: 45; p value = 0.973), indicating that both data fit the Poisson model well. Thus, the adjusted incidence rate ratio (IRR) was calculated and its 95.0% CI for the difference in incidence density of CVC-BSIs among the investigated periods. P values < 0.05 were considered statistically significant. In addition, a descriptive analysis of the variables related to the patient with CVC-BSIs was carried out (total and ICU).

The study was approved by the Ethics Committee in Tropical Diseases Hospital Research Dr. Anuar Auad, protocol n. 011/2012, and all ethical and legal principles were considered under Resolution n. 466/2012 of the National Health Council [22].

## 3. Results

The results of this study are presented by ICU.  Table 1 shows the variables and indicators related to the bundle in the adult and paediatric ICU. In adult ICU, a total of 2.282 applications-days of bundle was observed, resulting in an application rate of 89.8% in the postintervention period. Furthermore, there was an overall compliance of 85.6%. The item with the lowest application in the adult ICU was Item 4.

In pediatric ICU, there was a total of 438 applications-days, resulting in bundle application rate of 54.1% and full compliance of 4.3. Items 1 and 4 presented a low application rate in this ICU (Table 1).

In the adult ICU during the study period, we observed a total of 11,446 patients-days, 9,387 CVC-days, and an overall utilization rate of 82.0% CVC. A higher CVC usage fee in the preintervention period compared to the postintervention period (85.6% vs. 73.6%; p value < 0.001). We believe that the change in the patient profile in adult ICU (with decreased severity in the postintervention period) is responsible for the decrease of CVC usage fee. Still, there was a decrease in the number of cases of HAIs, ranging from 270 in the preintervention phase to 77 in the postintervention period. The overall incidence density of HAIs was 30.3 per 1,000 patient-days (95.0% CI: 27.3 to 33.6). A reduction in HAIs density per 1,000 patients-days between the periods was found (p value < 0.001) (Table 2).

It was also observed in the adult ICU that the occurrence of 32 cases of CVC-BSIS throughout the investigation period (25 in the preintervention period and 7 in the postintervention period). In the preintervention period, there was an incidence density of CVC-BSIs per 1,000 CVC-days of 3.65 (95.0% CI: 2.47 to 5.38). Despite the reduction in the absolute number of cases of CVC-BSIs, after the implementation of the bundle (after intervention), there was no significant reduction in the incidence density (IRR: 0.754; 95.0% CI: 0.349 to 1.621; p value = 0.469) (Table 2).

In the pediatric ICU, a total of 3.791 patients-days and 2.078 CVC- days were observed, resulting in an overall rate of use of CVC of 54.8%. It was found that the CVC usage fee significantly increased in the analyzed periods (p < 0.001). In this unit, there were a total of 51 cases of HAIs (density of 13.5 per 1,000 patients-days; 95.0% CI: 10.3 to 17.6). In addition, the incidence density of HAIs was reduced in the postintervention period (p value < 0.001) (Table 3).

During the study period, there was an overall per 1,000 CVC-days of 3.36 (95.0% CI: 1.63-6.93) in the pediatric ICU. In the preintervention period, there was an incidence density of CVC-BSIs of 3.15 by 1,000 CVC-days (95.0% CI: 1.22 to 8.07). There was no significant reduction in the incidence density of CVC-BSIs between the periods (IRR: 1.148; 95.0% CI: 0.314 to 4.193; p value = 0.834) obtained in fitted Poisson model (Table 3).

The characterization of these patients is presented in Table 4. It is noteworthy that most patients who developed CVC-BSIs in adult ICU were male (78.1%), while in the pediatric ICU they were female. In general, the main diagnosis in patients with CVC-BSIs was AIDS (48.7%), followed by tuberculosis (12.8%). The median length of hospital stay and CVC use was 39.5 days and 10.5 days, respectively.

Table 5 shows the characterization of the microorganisms identified in culture for diagnosis of CVC-BSIs. Most (61.8%) of the causative agents of CVC-BSIs in the institution were Gram-negative, with a predominance of* Pseudomonas aeruginosa *(28.2 %). Gram- positive ones accounted for 30.8% of the isolated microorganisms, highlighting* Staphylococcus aureus*. Fungi accounted for 10.3% of microorganisms, the most prevalent non*-albicans Candida*.

## 4. Discussion

Currently, the CVC-BSIs control has been the subject of national and international targets [17, 23]. The reduction of these infections is feasible and possible, since its occurrence is directly related to adoption of safe practice and protocol compliance, including the systematic use of bundles of prevention [24]. However, even well established in the practice, great are the challenges and constant is the quest for membership of professional best practice. There are few published studies on the evaluation of bundles in reducing CVC-BSIs in Latin America. This research adds to the literature about the effect of these strategies in rates CVC-BSIs rates in Brazil. The results showed that, even after implementation of bundles of prevention, significant reduction in incidence density of CVC-BSIs did not occur in both units assessed.

This investigation found an incidence density of CVC-BSIs per 1,000 CVC-days of 3.4 in the adult ICU, index below percentile 90 of Brazilian ICUs (11.8 CVC-BSIs per 1,000 CVC-days) [6] and higher than the American ICUs (2.8 CVC-BSIs per 1,000 CVC-days) [3]. Similarly, in the pediatric ICU, the incidence of overall incidence density was 3.36 CVC-BSIs per 1,000 CVC-days, rate below percentile 90 in pediatric ICU in Brazil in 2014 (14.2 CVC-BSIs per 1,000 CVC-days) [6] and higher than that found in pediatric ICU USA (2.0 CVC-BSIs per 1,000 CVC-days) [3].

Regarding the CVC utilization rate in the adult ICU, most patients used the device for most of the length of stay, although this rate shows lower postintervention period. However, in the pediatric ICU it was found that this ratio significantly increased in the postintervention period. Regardless of these differences, there was high use rate of this device during the period analyzed in both units. The utilization rates of CVC in adult and pediatric ICU are above the percentile of 75.0% of American hospitals evaluated by NHSN [3], probably reflecting the greater severity of patients admitted to the institution under study in relation to those hospitals that integrate the NHSN system. The CVC utilization rate reveals the degree of exposure to BSIs. Mesiano and Merchan-Hamann [25] point out that the maintenance of vascular access for a long time and with greater frequency of use results in increased infections related to that device.

Poisson regression models showed no significant reduction in the incidence density of CVC-BSIs in adult and pediatric ICUs after implementation of the bundle (after intervention) (p value > 0.05). This corroborates with other studies conducted in different geographical locations that have shown no significant reduction of infections after implementation of ICU prevention packages [26–29]. In USA, a randomized clinical trial in ICU of 60 hospitals also found no significant reduction after application of preventive bundles (2.42 to 2.73 CVC-BSIs per 1,000 CVC-days; p value = 0:59) [29]. In Taiwan, a study conducted in two ICUs found a similar rate of BSIs between periods of preintervention and after systematic implementation of bundles (1.58 to 1.06 CVC-BSIs per 1,000 CVC-days; p value = 0:31) [28]. In Spain, a study conducted in a university hospital found no reduction in pre- and post-application bundles (5.5 to 3.8 CVC-BSIs per 1,000 CVC-days; p value = 0.49) [27]. In Brazil, a study conducted by Wolf et al. [25] in the ICU of São Paulo showed that, even after bundle implementation, no significant reduction in incidence density of CVC-BSIs (20 to 11 CVC-BSIs per 1,000 CVC- days; p value = 0.07) [26].

The studies did not identify reduction in the incidence density of CVC-BSIs after bundles application emphasizing that their use in isolation does not bring decrease in infections, requiring a multidisciplinary approach and to consider the epidemiological profile of the institution and focused active leaders in continuous improvement processes [26–29]. In addition, factors such as high rate of use of CVC, low full compliance bundles application [26], low adherence to bundle and constant vigilance are factors that can decrease the effectiveness of intervention strategies. In fact, in this study, total compliance in the pediatric ICU and especially for Item 4 “assessment for early catheter removal” was very low, which contributed to the absence of significant reduction.

In the present study, there was a greater proportion of Gram-negative than Gram-positive microorganisms, unlike most studies conducted in North America that show a higher frequency of Gram-positivity in CVC-BSIs [30–32]. However, it corroborates with other studies previously published in several countries and regions [32–35]. In fact, studies in Latin America, such as Brazil, have shown a higher prevalence of Gram-negativity in CLABSI compared to American studies. Investigations such as SCOPE (Surveillance and Control of Pathogens of Epidemiological Importance) [36] and EPIC II (Extended Prevalence of Infection in Intensive Care) [32] show this difference. These studies discuss the possibility of a climate influence. [32, 36]. As Brazil is a tropical country, it has a warmer climate than in USA and some studies show a higher prevalence of Gram-negative summer/spring infections than in the autumn/winter where there would be more Gram-positive infections [37]. Another possibility would be a higher proportion of infections secondary to lung and urinary tract infections than in American studies [36].

This study has some limitations. First, in retrospective analyses there is no possibility of reporting bias with the inability to control confounding variables (lack of information). Second data as catheter insertion site and other risk factors of patients with CVC-BSIs were not subject to collection by the lack of information in the source data, data that could explain the lack of reduction in incidence density of CVC-BSIs. Thirdly, the number of new cases of CLABSIs in the postintervention period was very small in both ICUs (adult and pediatric). This may have diminished the power of the study to verify statistical differences. Other studies, with larger samples and in several hospitals, are needed. Fourth, the analysis period after intervention period was relatively short to evaluate the effect of long-term bundle. Finally, the results cannot be generalized to all ICUs because they are only considered units of an institution.

## 5. Conclusion

In conclusion, there was no significant reduction in the incidence density CVC-BSIs in adult ICU (p value = 0.469) and pediatrics (p = 0.834) after implantation of the bundle of prevention. There was an increase of CVC utilization rate in both ICUs and low total bundle compliance in the pediatric ICU in the postintervention period, which indicate bias application of care for CVC-BSIs prevention.

The results of this study show a need to reassess the strategy, as well as continuous training for the application bundle and measurement of compliance with discussion of process indicators with the care team. It is the multidisciplinary team treating the patient that takes responsibility in this chain of transmission, adhering to the protocols of prevention. Managers remain with the implicit responsibility to manage the processes, train professionals, and provide favorable conditions for the implementation of preventive measures in health care practice. The implications for the management deserve attention, since joining the bundle of practice is based on actions that do not require additional costs, but the adoption of preventive measures by professionals, since health institutions are already well structured with respect to human resources and materials. The findings of this study suggest managers periodically investigate the indicators of the CVC application process (bundles) and the occurrence of CVC-BSIs to identify the root causes and implement new preventive measures and evaluation of bundles of prevention. Further studies are needed to evaluate the effect of bundle prevention of CVC-BSIs long term in Brazil.

## Figures and Tables

**Table 1:** Variables and bundle indicators in adult and pediatric ICUs. Central Brazil. 2014-2015.

Variables and indicators bundle	Adult ICU	Pediatric ICU
Applications-day	2.282	438
Bundle application rate (%)	89.8	54.1
Item 1	60.2	48.6
Item 2	99.8	99.8
Item 3	99.8	95.9
Item 4	88.1	5.7
Total Compliance	85.6	4.3

ICU: Intensive Care Unit.

**Table 2:** Evaluated variables and indicators in the adult ICU. Central Brazil. 2012-2015.

Variables and indicators	All	Periods		*p value*
		Preintervention_ _^a^	Postintervention_ _^b^	
Number of patients-days	11.446	7.995	3.451	-
Number of episodes of HAIs	347	270	77	-
Incidence density of HAIs (95.0% CI)	30.3 (27.3-33.6)	33.8 (30.0-38.0)	22.3 (17.9-27.8)	< 0.001_ _^c^
CL utilization (%) (95.0% CI)_ _^b^	82.0 (81.3-82.7)	85.6 (84.9-86.4)	73.6 (72.1-75.1)	< 0.001_ _^c^
Number of new cases of CLABSIs	32	25	7	-
Number of deaths from CLABSIs	1	7	3	-
Lethality with CVC-BSIs (%) (95.0% CI)_ _^b^	31.3 (18.0-49.6)	28.0 (14.3-47.6)	42.9 (15.8-75.0)	0.459_ _^c^
CVC-days	9.387	6.847	2.540	-
Incidence density of CVC-BSIs (95.0%CI)_ _^b^	3.40 (2.41-4.80)	3.65 (2.47-5.38)	2.75 (1.33-5.47)	0.469_ _^d^

95.0% CI: 95.0% Confidence Interval; a. Preintervention period: January 2012 to October 2014; b. Postintervention period: November 2014 to December 2015; c. Wald Statistics; d. Wald Statistics obtained in fitted Poisson model. CL: Central Line; CVC: Central venous catheter; CVC-BSIs: Central venous catheter-associated bloodstream infections; HAIs: Healthcare-associated infections; ICU: Intensive Care Unit.

**Table 3:** Evaluated variables and indicators in the pediatric ICU. Central Brazil. 2012-2015.

Variables and indicators	All	Periods		*p value*
		Preintervention_ _^a^	Postintervention_ _^b^	
Number of patients-days	3.791	2.575	1.216	-
Number of episodes of HAIs	51	35	16	-
Incidence density of HAIs (95.0% CI)	13.5(10.2-17.6)	13.6 (9.8-18.8)	12.2 (8.1-21.3)	0.783_ _^c^
CL utilization (%) (95.0% CI)_ _^b^	54.8 (53.2-56.3)	49.6 (47.7-51.6)	66.5 (63.8-69.1)	< 0.001_ _^c^
Number of new cases of CLABSIs	7	4	3	-
Number of deaths from CLABSIs	-	-	-	-
Lethality with CVC-BSIs (%) (95.0% CI)_ _^b^	-	-	-	-
CVC-days	2.078	1.269	809	-
Incidence density of CVC-BSIs (95.0%CI)_ _^b^	3.36(1.63-6.93)	3.15(1.22-8.07)	3.70(1.26-10.84)	0.834_ _^d^

95.0% CI: 95.0% Confidence Interval; a. Preintervention period: January 2012 to August 2014; b. Postintervention period: September 2014 to December 2015; c. Wald statistics; d. Wald Statistics obtained in fitted Poisson model. CL: Central Line; CVC: Central venous catheter; CVC-BSIs: Central venous catheter-associated bloodstream infections; HAIs: Healthcare-associated infections; ICU: Intensive Care Unit.

**Table 4:** Characterization of patients with CVC-BSIs. Central Brazil. 2012-2015.

Variables	All (n = 39)	Adult ICU (n = 32)	Pediatric ICU (n = 7)
*Age (yeas) (Median; IIQ)*	44.5 (23.0)	46.5 (18.0)	5.0 (8.0)
*Length of stay (days) (Median; IIQ)*	39.5 (38.0)	42.0 (44.0)	33.0 (30.0)
*CL usage time (days) (Median; IIQ)*_ _^c^	10.5 (8.0)	10.0 (8.0)	12.0 (8.0)
*Sex*			
Male	27 (69.2)	25 (78.1)	2 (28.6)
Female	12 (30.8)	7 (21.9)	5 (71.4)
*Diagnoses*			
AIDS	19 (48.7)	19.0 (59.4)	-
Tuberculosis	5 (12.8)	5 (15.6)	-
Viral hepatitis	1 (2.6)	1 (3.1)	-
Leishmaniasis	2 (5.1)	2 (6.2)	-
Dengue	1 (2.6)	-	1 (14.3)
Leprosy	1 (2.6)	1 (3.1)	-
Meningitis	2 (5.1)	1 (3.1)	1 (14.3)
Other lung infections	2 (5.1)	-	2 (28.6)
Tetanus	1 (2.6)	1 (3.1)	-
Others	6 (15.4)	3 (9.4)	3 (42.9)

AIDS: Acquired immunodeficiency syndrome; ICU: Intensive Care Unit.

**Table 5:** Characterization of the microorganisms identified in culture for diagnosis of CVC-BSIs.

Microorganisms	All (n = 39)	Adult ICU (n = 32)	Pediatric ICU (n = 7)
Gram-positive	12 (30.8)	10 (31.2)	2 (28.6)
*Staphylococcus aureus*	6 (15.4)	2 (12.5)	2 (28.6)
*Staphylococcus epidermidis*	3 (7.7)	3 (9.4)	-
*Staphylococcus coagulase negativos*	1 (2.6)	1 (3.1)	-
*Enterococcus faecalis*	2 (5.1)	2 (6.2)	-
*Streptococcus salivarius*	1 (2.6)	1 (3.1)	-
Gram-negative	24 (61.8)	20 (62.5)	4 (57.1)
*Pseudomonas auruginosa*	11 (28.2)	8 (25.0)	3 (42.9)
*Acinetobacter *spp.	5 (12.8)	5 (15.6)	-
*Enterobacter *spp.	5 (12.8)	3 (9.4)	2 (28.6)
*Klebsiella pneumoniae*	5 (12.8)	5 (15.6)	-
*ESBL Klebsiella*	1 (2.6)	1 (3.1)	-
*Achromobacter xylosoxidans*	1 (2.6)	1 (3.1)	-
Fungi	4 (10.3)	3 (9.4)	1 (14.3)
*Candida albicans*	2 (5.1)	1 (3.1)	1 (14.3)
*Candida não albicans*	3 (7.7)	3 (9.4)	-

ICU: Intensive Care Unit.

## Data Availability

The data will be made available by the corresponding author if requested.
